# Development and internal evaluation of a clinical prediction model for blood lead re-elevation in lead poisoning patients: a retrospective cohort study

**DOI:** 10.3389/ftox.2026.1829277

**Published:** 2026-07-30

**Authors:** Jia Wu, Xuanqi Zhang, Sijie Hu, Xi Li, Tianwen Yang, Jie Yao, Yingjie He, Jian Gao, Yongyi Wang, Youchun Lei

**Affiliations:** 1 School of Medical Information and Engineering, Southwest Medical University, Luzhou, China; 2 Department of Gastroenterology, The Xinqiao Hospital of Army Medical University, Chongqing, China; 3 Foundation Department, Chongqing Medical and Pharmaceutical College, Chongqing, China; 4 Department of Gastroenterology, The First Affiliated Hospital of Chongqing Medical and Pharmaceutical College, Chongqing, China; 5 Occupational Disease and Poisoning Department, The First Affiliated Hospital of Chongqing Medical and Pharmaceutical College, Chongqing, China; 6 Department of Gastroenterology, The Second Affiliated Hospital, Chongqing Medical University, Chongqing, China; 7 Department of Chongqing Key Laboratory of Prevention and Treatment for Occupational Diseases and Poisoning, The First Affiliated Hospital of Chongqing Medical and Pharmaceutical College, Chongqing, China

**Keywords:** blood lead re-elevation, lead poisoning, machine learning, prediction model, risk stratification

## Abstract

**Background:**

Blood lead can rise again after chelation therapy for lead poisoning, but few tools identify which patients are at highest risk. We developed and internally evaluated a model to predict this re-elevation from routinely collected clinical data.

**Methods:**

We conducted a retrospective cohort study of 163 patients hospitalized for lead poisoning at Chongqing Poison Control Center from 2014 to 2022. Blood lead re-elevation was ascertained from re-admission for lead poisoning after an initial response to chelation therapy. Logistic regression, random forest, and support vector machine models were evaluated. The dataset was randomly divided into training (70%, n = 114) and testing (30%, n = 49) sets. Model performance was assessed using area under the receiver operating characteristic curve (AUC), accuracy, precision, recall, calibration, and decision curve analysis.

**Results:**

Among 163 patients, 59 (36.2%) experienced blood lead re-elevation. The cohort comprised 44 children (27.0%) and 119 adults (73.0%), and 111 patients (68.1%) were male. Pediatric patients had a higher re-elevation rate than adults (52.3% vs. 30.3%, p = 0.016). The random forest model showed the best internal test-set performance, with an AUC of 0.849, accuracy of 83.7%, precision of 77.8%, and recall of 77.8%. Important predictors included blood lead-to-season ratio, baseline-to-follow-up blood lead ratio, serum calcium-to-blood lead ratio, blood lead-to-hemoglobin ratio, and hemoglobin-to-AST ratio.

**Conclusion:**

An internally evaluated model can identify patients at higher risk of blood lead re-elevation after chelation therapy and could guide risk-based follow-up. External validation is needed before clinical use.

## 1 Introduction

Lead is a toxic heavy metal that has been widely used in battery manufacturing, gasoline, paint, pigments, glazes, plastics, ceramics, and water pipes due to its high density, low melting point, easy processing, and good corrosion resistance (Balza et al., 2025; Bridbord and Hanson, 2009; Lanphear et al., 2024; Levin et al., 2021). Lead contamination is widespread in human production and daily life, causing recurrent lead poisoning in children and adults, which places a substantial burden on families and society (Ruckart et al., 2023; Larsen and Sánchez-Triana, 2023).

Most patients with lead poisoning remain asymptomatic, making early detection and monitoring challenging. Blood lead re-elevation is a clinical concern because of lead’s long biological half-life and the potential for cumulative organ damage. Our research team conducted a retrospective analysis of lead poisoning cases at the Chongqing Poison Control Center (Chongqing Institute of Occupational Disease Prevention and Treatment) and found that pediatric patients experience blood lead re-elevation more often than adults (52.3% vs. 30.3%); age is therefore an important risk factor. Due to the lack of specific clinical manifestations in patients with mild to moderate lead poisoning, many patients with recurrent elevated blood lead levels have poor adherence to follow-up care, failing to follow medical advice for timely blood lead monitoring after discharge from chelation therapy. This poor compliance results in prolonged periods of undetected re-elevated blood lead levels, leading to progressive accumulation of damage to the nervous system, cognitive function, and multiple organ systems (Ye and Wong, 2006; Liang et al., 2022; Hou et al., 2025).

**FIGURE 1:**
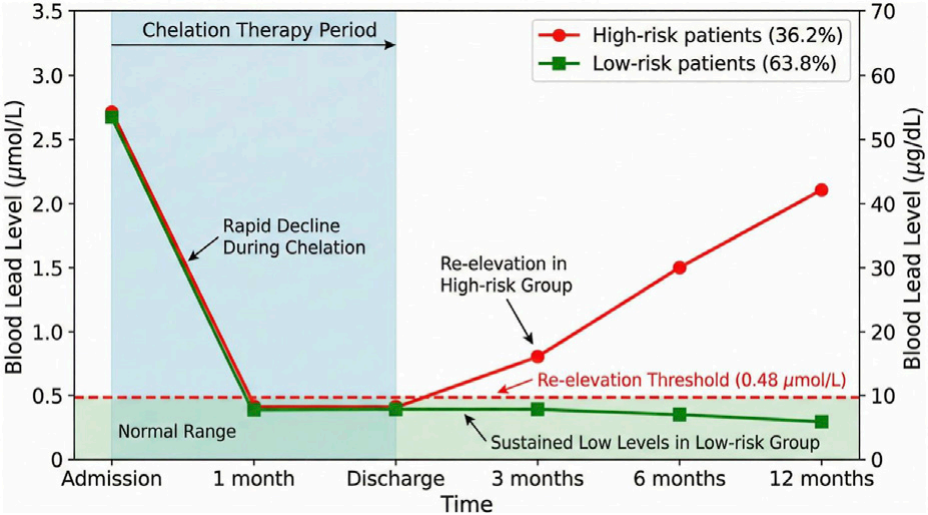
Schematic illustration of blood lead dynamics and re-elevation following chelation therapy. Blood lead levels at diagnosis (median 2.7 μmol/L) decrease following chelation therapy to normal levels (<0.48 μmol/L or <100 μg/L). In some patients blood lead later rises again as lead is mobilized from bone stores or through continued environmental exposure; re-elevation, operationally ascertained as re-admission for lead poisoning, occurred in 36.2% of this cohort. The dashed line marks the normal threshold (0.48 μmol/L, 100 μg/L).

Lead enters the human body primarily through the gastrointestinal tract and respiratory tract, and the skin can also absorb some organic lead compounds (Zhao et al., 2024). Lead enters the blood and binds to red blood cells, maintaining equilibrium between cell-associated lead and plasma lead. In bones and teeth, lead can be stored for decades (de Souza et al., 2018). Because of these toxicokinetics, lead can remain in the body for decades after exposure, and blood lead can rebound after chelation as lead is mobilized from bone stores. Some patients will experience recurrent blood lead elevation (Figure 1), and solving lead poisoning requires continuous treatment to eliminate bone lead content to avoid endogenous exposure (World Health Organization, 2019).

Lead toxicity poses particularly severe risks to children due to their greater absorption, increased permeability of the blood-brain barrier, and ongoing neurodevelopment. There is no recognized safe blood lead threshold in children: even concentrations below 50 μg/L have been associated with subclinical neurodevelopmental deficits, including measurable reductions in IQ and attention (Rawat et al., 2022; World Health Organization, 2024; Council on Environmental Health, 2016; Canfield et al., 2005; Grandjean and Landrigan, 2014), whereas overt and potentially irreversible lead encephalopathy (seizures, coma) generally occurs at much higher concentrations (≥700 μg/L) (National Health and Family Planning Commission of China, 2006). Lead in adults can damage the nervous system, circulatory system, gastrointestinal system, and others (Wani et al., 2015). In women, lead poisoning can cause miscarriage (Omeljaniuk et al., 2018), low birth weight infants, premature infants, and developmental problems in children (Fisher et al., 2023). In children, lead poisoning can interfere with learning ability, impair memory, reduce IQ, and interfere with growth and development. In addition, lead poisoning can also cause speech, hearing and nerve conduction disorders, hyperactivity, intestinal discomfort, constipation, vomiting, weight loss, and muscle pain (Al Osman et al., 2019).

Recurrent blood lead elevation places a continuing burden on patients, who require repeated chelation therapy and continuous micronutrient supplementation. Treatment interruption can cause irreversible damage to multiple organ systems, particularly children’s developing nervous systems and adults’ cardiovascular and renal systems. Poor compliance with follow-up visits creates a critical window where blood lead re-elevation can occur silently, resulting in cumulative damage affecting organ function and quality of life.

In current practice, the management of lead poisoning follows a stepwise protocol. The first step is identification and removal of the lead source, including occupational exposure, the inappropriate use of traditional remedies, and environmental or hand-to-mouth exposure in children (National Health and Family Planning Commission of China, 2006). Chelation therapy is then guided by severity: at our center, calcium disodium edetate (CaNa2EDTA) is the principal chelating agent, given in courses of 3–5 days separated by drug-free intervals, with the dose and number of courses individualized to severity (in children, 25 mg/kg/day by intravenous infusion) (National Health and Family Planning Commission of China, 2006; GBZ 37-2015, 2015). Adjuvant therapy is used selectively, with reduced glutathione given when aminotransferases are elevated and sodium ferulate used mainly in adults with cardiovascular comorbidity. Because lead impairs the absorption and status of essential elements, nutritional support is routine, with supplementation of iron, calcium, zinc and vitamins C and D; iron deficiency is corrected before chelation, and iron, zinc and calcium are rechecked after repeated courses (Levander, 1979; Mahaffey, 1981). Mechanistically, calcium competes with lead for intestinal transporters (TRPV6 and Ca2+-ATPase), iron competes for the divalent metal transporter DMT1, and zinc restores the activity of δ-aminolevulinic acid dehydratase, providing a biological basis for micronutrient repletion (Levander, 1979; Mahaffey, 1981; Zhang et al., 2024). After discharge, blood lead is rechecked approximately 4 weeks after the last course, and further treatment is determined by whether levels remain at or above the 250 μg/L (children) or 450 μg/L thresholds (National Health and Family Planning Commission of China, 2006).

Current clinical guidelines from the Centers for Disease Control and Prevention (CDC) and American Academy of Pediatrics (AAP) provide general recommendations for blood lead level monitoring and management, but critically lack individualized prediction tools for identifying patients at high risk of blood lead re-elevation (Irawati et al., 2022; Varela, 2023; Binns et al., 2007). This is an important gap in clinical practice, because the variation in re-elevation rates (52.3% in children vs. 30.3% in adults) calls for better risk stratification. Clinical physicians primarily rely on empirical judgment and baseline blood lead levels to determine follow-up frequency, which has limited predictive capability for identifying patients who will experience lead level rebound (Potash et al., 2020).

Follow-up after discharge is difficult in practice. Patients typically remain asymptomatic during early re-elevation stages, creating a false sense of recovery. Despite clear follow-up instructions, patients often miss scheduled monitoring because they have no visible symptoms, so elevated blood lead can go undetected and treatment is delayed.

A prediction model could address this by giving patients a clear, quantified estimate of their risk, which may improve adherence and reduce the harm from delayed detection.

Although several studies have applied machine learning approaches to predict initial blood lead elevation in community populations (Potash et al., 2020; Mulhern et al., 2022; Abhadiomhen et al., 2024), research specifically focused on blood lead re-elevation prediction following chelation therapy remains absent. Previous prediction models, such as those by Potash et al. (2020) and Mulhern et al. (2022), have successfully identified children at risk for initial elevated blood lead levels using demographic and environmental data. However, these models address a fundamentally different clinical question, predicting first-time elevation rather than post-treatment recurrence. The phenomenon of blood lead re-elevation, occurring in approximately 40% of children after chelation therapy due to lead mobilization from bone stores, represents a distinct clinical challenge that has not been addressed by existing prediction tools (Flora et al., 2012; Barrett et al., 2024). The heterogeneity of patient populations and the complex toxicokinetics of lead also make accurate prediction difficult with single biomarkers (Gwaltney‐Brant, 2025; Xu et al., 2018). The lack of explainable artificial intelligence applications in lead toxicology limits clinical acceptance and implementation of prediction models in routine practice (Lundberg and Lee, 2017; Unger and Kather, 2024; Berríos-Torres et al., 2017).

These models also differ from the present work in their inputs: they rely largely on environmental and sociodemographic variables, whereas a hospitalized cohort provides detailed clinical, laboratory and treatment data that may better capture the determinants of recurrence. Interpretable models that pair a single algorithm with feature-level explanation have proved useful for related clinical prediction tasks, including acute kidney injury in critically ill children (Hu et al., 2024) and postoperative delirium in older surgical patients (Song et al., 2024), and we adopt the same strategy for blood lead re-elevation.

Based on the clinical burden of blood lead re-elevation and the absence of prediction tools in current guidelines, we developed a clinical prediction model using routinely available clinical data. This study aimed to develop and internally evaluate a prediction model for blood lead re-elevation through machine learning approaches by analyzing a retrospective cohort of 163 patients (both pediatric and adult), identifying key clinical predictors, comparing multiple algorithms, and translating the final model into a practical decision support tool. To our knowledge, this research represents the first systematic approach to blood lead re-elevation prediction following chelation therapy, addressing a critical gap in lead poisoning management.

## 2 Methods

### 2.1 Study design and patients

This retrospective cohort study was conducted at the Chongqing Poison Control Center (Chongqing Institute of Occupational Disease Prevention and Treatment) from January 2014 to December 2022. The study protocol was approved by the institutional review board, and informed consent was waived due to the retrospective nature of the analysis. All procedures were performed in accordance with the Declaration of Helsinki and relevant guidelines for clinical research.

**FIGURE 2:**
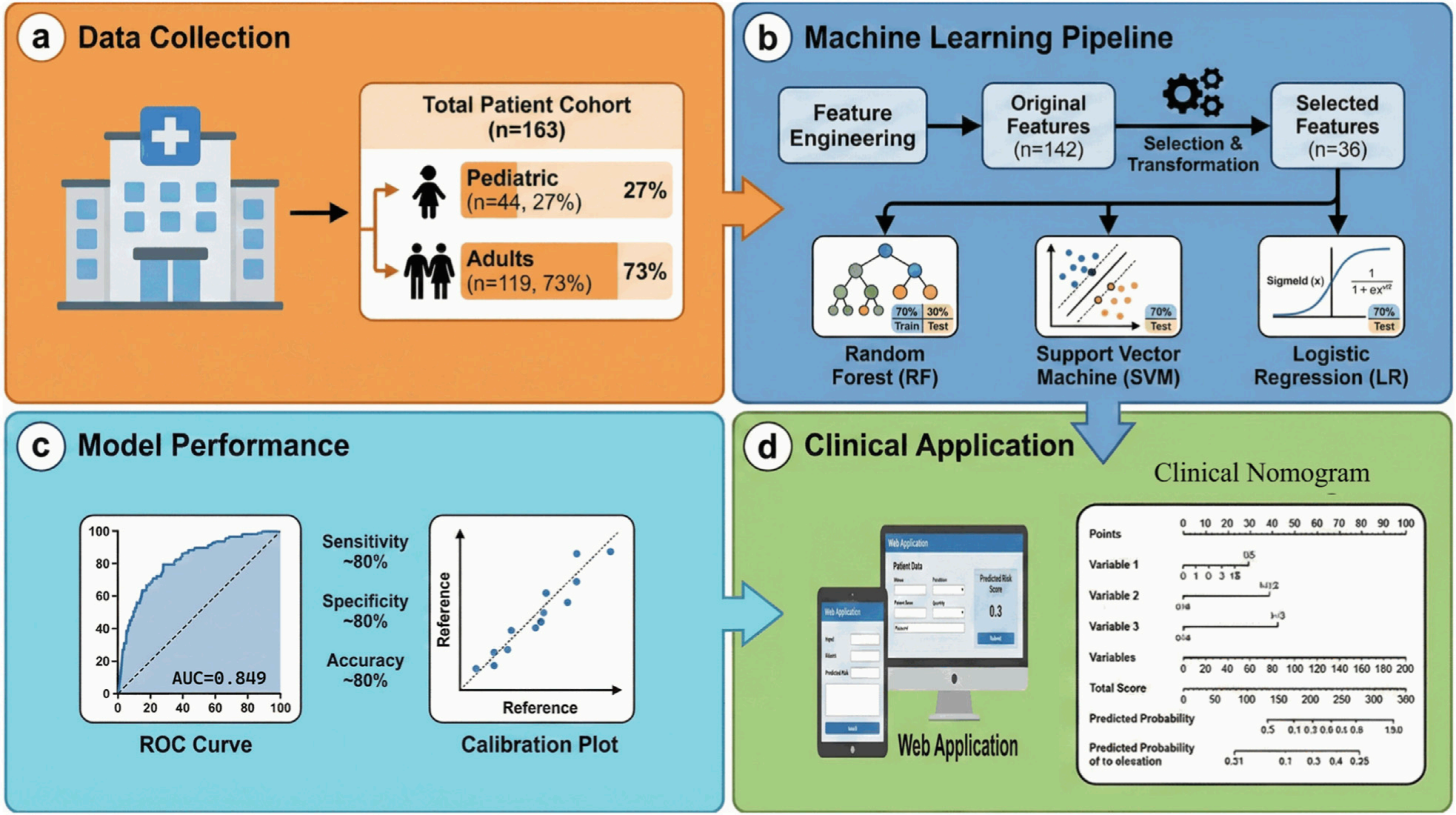
Overview of the study design and machine learning workflow. **(a)** Patient cohort: 163 lead poisoning patients from Chongqing Poison Control Center (2014–2022), divided into training (70%, n = 114) and testing (30%, n = 49) sets. **(b)** Feature engineering: 142 clinical variables transformed into 36 optimized predictors through medical ratio features and interaction terms. **(c)** Model development: comparison of Random Forest, SVM, and Logistic Regression algorithms with hyperparameter optimization. **(d)** Clinical application: individualized risk estimation and a web-based prediction tool to support personalized follow-up strategies.

We included all patients who were hospitalized for lead poisoning during the study period, including both pediatric (age ≤18 years) and adult patients for broad population coverage. Inclusion criteria were confirmed diagnosis of lead poisoning based on blood lead levels ≥0.96 μmol/L (≥200 μg/L) for mild poisoning according to Chinese “Guidelines for Prevention and Management of Childhood Elevated Blood Lead Levels and Lead Poisoning” (National Health and Family Planning Commission of China, 2006), complete clinical records including baseline characteristics and laboratory results, and available follow-up data for at least 6 months post-discharge. Exclusion criteria included patients with incomplete medical records, concurrent acute poisoning from other heavy metals, patients who died during the initial hospitalization, and patients lost to follow-up within 30 days post-discharge. The study design and machine learning workflow are illustrated in Figure 2.

Children and adults differ in lead toxicokinetics: children absorb a larger fraction of ingested lead (approximately 40%–50% versus 10%–15% in adults), have a more permeable blood-brain barrier, and undergo faster bone turnover, so blood lead falls more quickly during chelation in adults (O'Flaherty, 1998; Rabinowitz, 1991; Barry, 1975; Halmo and Nappe, 2023). The mechanisms that drive re-elevation after treatment are nonetheless shared between the two groups, namely, endogenous release of skeletal lead and its redistribution to saturable erythrocyte binding sites, together with re-exposure, nutritional status and adherence to follow-up (O'Flaherty, 1998; Rabinowitz, 1991; Barry, 1975). We therefore modeled children and adults together, including age and sex as predictors so that age-dependent effects could still be captured; the development of separate age-specific models is left to future, larger studies.

### 2.2 Data collection

Clinical data were extracted from electronic medical records by trained personnel using a standardized data collection form. The extracted variables included demographic and clinical characteristics such as age at admission, gender, clinical symptoms and signs at presentation, and medical history. Lead exposure assessment covered source and route of exposure, duration of exposure, and family history of lead exposure. Laboratory parameters included blood lead levels at admission and during follow-up, complete blood count, metabolic panel, and nutritional markers. Treatment details included chelation therapy regimen, adjuvant medications, length of hospital stay, and response to treatment. The baseline characteristics and clinical variables are summarized in Table 1, while laboratory parameters and treatment variables are presented in Table 2.

**TABLE 1:** Patient baseline characteristics and clinical variables.

Characteristic	Overall (n = 163)	No Re-elevation (n = 104)	Re-elevation (n = 59)	p-value
Demographics and clinical presentation				
Age (years), median (IQR)	46.0 (10.5–55.0)	47.5 (27.5–54.0)	43.0 (3.5–58.5)	0.468
Male gender, n (%)	111 (68.1)	72 (69.2)	39 (66.1)	0.813
Pediatric patients (≤18 years), n (%)	44 (27.0)	21 (20.2)	23 (39.0)	0.016
Blood lead level at admission (μmol/L), median (IQR)	2.7 (2.0–3.6)	2.9 (2.4–3.8)	2.2 (1.5–2.9)	<0.001
Length of hospital stay (days), median (IQR)	16.0 (8.5–26.0)	21.0 (11.0–27.0)	12.0 (6.5–18.5)	<0.001
Symptomatic at admission, n (%)	97 (59.5)	74 (71.2)	23 (39.0)	<0.001
Clinical symptoms				
Abdominal pain, n (%)	67 (41.1)	57 (54.8)	10 (16.9)	<0.001
Abdominal distension, n (%)	40 (24.5)	34 (32.7)	6 (10.2)	0.003
Nausea/vomiting, n (%)	17 (10.4)	16 (15.4)	1 (1.7)	0.013
Decreased appetite, n (%)	29 (17.8)	25 (24.0)	4 (6.8)	0.011
Constipation, n (%)	24 (14.7)	21 (20.2)	3 (5.1)	0.017
Fatigue, n (%)	40 (24.5)	35 (33.7)	5 (8.5)	<0.001
Dizziness, n (%)	24 (14.7)	20 (19.2)	4 (6.8)	0.054
Hematologic parameters				
White blood cell count (×10^9/L), median (IQR)	6.3 (5.0–8.0)	6.4 (5.1–7.9)	6.0 (5.0–8.9)	0.945
Neutrophils (%), median (IQR)	63.0 (48.5–70.5)	64.7 (51.6–73.2)	60.0 (41.5–65.5)	0.005
Lymphocytes (%), median (IQR)	28.1 (21.1–42.2)	27.3 (17.9–37.4)	30.6 (23.9–49.5)	0.014
Red blood cell count (×10^12/L), median (IQR)	4.4 (3.5–4.9)	4.2 (3.2–4.9)	4.4 (4.0–4.8)	0.120
Hemoglobin (g/L), median (IQR)	124.0 (105.2–134.8)	115.5 (95.0–137.2)	129.0 (118.2–134.0)	0.006
Mean corpuscular volume (fL), median (IQR)	89.3 (84.2–94.0)	89.3 (85.1–93.4)	89.8 (79.5–95.3)	0.968

**TABLE 2:** Laboratory parameters and treatment variables.

Characteristic	Overall (n = 163)	No re-elevation (n = 104)	Re-elevation (n = 59)	p-value
Laboratory parameters				
Platelet count (×10^9/L), median (IQR)	210.0 (172.2–273.8)	214.0 (177.8–273.2)	201.5 (170.0–276.8)	0.484
Serum potassium (mmol/L), mean ± SD	4.1 ± 0.5	4.1 ± 0.5	4.2 ± 0.4	0.357
Serum sodium (mmol/L), median (IQR)	139.0 (137.1–140.9)	138.7 (136.8–140.9)	139.5 (138.0–141.1)	0.139
Serum calcium (mmol/L), median (IQR)	2.3 (2.3–2.5)	2.3 (2.2–2.4)	2.4 (2.3–2.5)	<0.001
Alanine aminotransferase (U/L), median (IQR)	22.3 (15.5–39.9)	23.4 (17.6–49.0)	18.9 (13.1–28.9)	0.003
Aspartate aminotransferase (U/L), median (IQR)	30.5 (24.2–37.0)	31.6 (24.2–46.4)	29.1 (24.2–33.7)	0.058
Alkaline phosphatase (U/L), median (IQR)	86.2 (68.8–196.9)	86.1 (69.3–175.8)	88.8 (68.1–225.6)	0.336
Total protein (g/L), mean ± SD	67.1 ± 5.2	66.6 ± 5.0	67.9 ± 5.5	0.152
Albumin (g/L), median (IQR)	44.3 (42.0–45.9)	44.3 (41.9–45.8)	44.5 (42.1–46.3)	0.540
Serum creatinine (μmol/L), median (IQR)	65.5 (42.1–77.0)	68.5 (50.1–80.2)	60.1 (29.0–72.3)	0.016
Treatment and follow-up				
Total chelation therapy dose (g), median (IQR)	7.0 (3.0–13.0)	9.0 (4.0–14.0)	4.0 (1.6–7.0)	<0.001
Follow-up blood lead monitoring times, median (IQR)	0.0 (0.0–2.0)	0.0 (0.0–1.0)	0.0 (0.0–7.0)	0.012
Comorbidities and complications				
Attention deficit, n (%)	20 (12.3)	11 (10.6)	9 (15.3)	0.531
Memory impairment, n (%)	9 (5.5)	6 (5.8)	3 (5.1)	1.000
Family history of lead exposure, n (%)	5 (3.1)	1 (1.0)	4 (6.8)	0.110
Occupational lead exposure, n (%)	56 (34.4)	41 (39.4)	15 (25.4)	0.102
Toxic hepatopathy, n (%)	34 (20.9)	27 (26.0)	7 (11.9)	0.054
Anemia, n (%)	31 (19.0)	26 (25.0)	5 (8.5)	0.018
Adjuvant medications				
Use of reduced glutathione, n (%)	108 (66.3)	79 (76.0)	29 (49.2)	<0.001
Use of sodium ferulate, n (%)	9 (5.5)	7 (6.7)	2 (3.4)	0.589
Use of antibiotics, n (%)	9 (5.5)	9 (8.7)	0 (0.0)	0.049
Treatment outcome				
Normal blood lead at last follow-up, n (%)	66 (40.5)	46 (44.2)	20 (33.9)	0.260

IQR, interquartile range; SD, standard deviation.

Blood lead was measured in the hospital’s clinical laboratory by graphite-furnace atomic absorption spectrometry (PerkinElmer AAnalyst 800). Measurements followed the Chinese occupational-health standard GBZ/T 316.1–2018 (“Determination of lead in blood, Part 1: graphite furnace atomic absorption spectrometry”); before 1 January 2019 the superseded standard WS/T 20–1996 was used, with no change of instrument. The limit of detection was 2.6 μg/L and the limit of quantification 10.0 μg/L (for a 0.1 mL sample diluted ten-fold). Quality assurance comprised internal quality-control samples (obtained from the National Center for Occupational Health and Poison Control, China CDC) analyzed with every batch, and annual external proficiency testing organized by the China CDC and the Chongqing quality-control center, which the laboratory passed throughout the study period (GBZ/T 316.1-2018, 2018).

The baseline characteristics showed several patterns. The re-elevation group had higher pediatric representation (39.0% vs. 20.2%, p = 0.016), lower initial blood lead levels (2.2 vs. 2.9 μmol/L, p < 0.001), fewer symptoms at admission (39.0% vs. 71.2%, p < 0.001), shorter hospital stays (12.0 vs. 21.0 days, p < 0.001), and lower chelation therapy doses (4.0 vs. 9.0 g, p < 0.001), differences that are largely explained by the higher proportion of children in the re-elevation group, who tend to present at lower blood lead with fewer symptoms and to receive lower absolute chelation doses; these are associations rather than evidence that under-treatment causes re-elevation.

### 2.3 Outcome definition

The primary outcome was blood lead re-elevation after an initial response to chelation therapy. Because post-discharge monitoring was sparse (Section 2.4), re-elevation was operationally ascertained from re-admission for lead poisoning, that is, a second or subsequent hospitalization of the same patient. Under the center’s admission criteria a patient is re-admitted only when blood lead has risen back to the diagnostic threshold for lead poisoning (≥0.96 μmol/L, ≥200 μg/L), so re-admission marks an objective, clinically confirmed re-elevation rather than a transient or subclinical fluctuation. For reference, blood lead below 0.48 μmol/L (100 μg/L) is regarded as normal and 0.48 μmol/L or above as elevated under the Chinese national grading principles (National Health and Family Planning Commission of China, 2006). The outcome was defined at the patient level (re-admitted versus not), with the need for repeat hospitalization as its operational expression.

Blood lead concentration also governs severity and the intensity of treatment under the relevant Chinese standards (GBZ 37–2015 for occupational poisoning in adults and the 2006 national grading principles for children) (National Health and Family Planning Commission of China, 2006; GBZ 37-2015, 2015). In a child, 300 μg/L (1.45 μmol/L) represents moderate poisoning, usually with few or only mild symptoms, and is managed by source removal, nutritional support and chelation; 800 μg/L (3.86 μmol/L) represents severe poisoning in a child and at least mild-to-moderate poisoning in an adult, is more often symptomatic (abdominal pain, anemia, impaired attention), and calls for more intensive chelation and management of complications. The clinical significance of re-elevation therefore varies across this range.

### 2.4 Feature engineering and data quality

We performed feature engineering to capture the pathophysiology of lead toxicity and re-elevation patterns. Features with more than 70% missing values were removed. Missing values in numerical features were handled using iterative imputation with 10 iterations (Laqueur et al., 2022), while categorical features were filled with mode values. Date-time features were decomposed into seasonal components and time differences to capture temporal patterns.

Records were considered usable when the key clinical and laboratory fields were present, mutually consistent and within physiologically plausible ranges, with all laboratory values obtained from quality-controlled hospital assays. Post-discharge monitoring of blood lead was sparse, at a median of zero follow-up measurements per patient, which reflects the poor adherence that motivated this study; the re-elevation outcome was therefore established from documented re-elevation and re-admission records rather than from continuous longitudinal measurement.

### 2.5 Medical feature engineering

Using knowledge of lead toxicity mechanisms, we constructed composite clinical features. We created lead toxicity severity indices by calculating blood lead-to-age ratios to capture developmental vulnerability in pediatric patients, where younger patients show higher susceptibility to lead toxicity effects. This ratio feature accounts for the inverse relationship between age and lead absorption rates in developing children.

To capture complex relationships between clinical parameters, we generated ratio features between key medical indicators in the dataset, creating over 100 interaction features between blood lead levels, hematological parameters (hemoglobin, red blood cell count, red cell indices), liver function markers (ALT, AST, bilirubin), serum calcium measurements, and follow-up monitoring variables. These interaction features reflect important pathophysiological relationships such as lead-calcium competitive inhibition, hematological toxicity patterns, and multi-organ system involvement. Polynomial interaction features were created for the most important clinical indicators using degree-2 polynomial transformations to capture non-linear dose-response relationships, resulting in 10 additional polynomial features. Advanced composite features included lead accumulation ratios computed between different measurement timepoints, anemia composite indices integrating multiple hematological parameters, and liver function abnormality indices combining liver enzyme measurements. Logarithmic transformations were applied to normalize highly skewed distributions of blood lead measurements and liver enzyme levels to improve model performance.

The candidate variables were chosen for established or biologically plausible links to lead toxicity. Lead inhibits δ-aminolevulinic acid dehydratase and ferrochelatase, producing anemia; in our prior clinical analysis of this population, hemoglobin was reduced in both age groups, the neutrophil percentage was higher in adults (consistent with chronic inflammatory stress) and the lymphocyte percentage was higher in children, supporting the inclusion of these hematologic indices (Wani et al., 2015; Al Osman et al., 2019). Hepatic involvement (reflected by ALT and AST) and renal involvement (reflected by creatinine) are recognized features of lead toxicity, the latter consistent with reported associations between heavy-metal exposure and chronic kidney disease (Jalili et al., 2021). Serum calcium was included because of calcium–lead competition during absorption and bone turnover, and antibiotic use was included as a marker of concurrent infection rather than a direct toxic mechanism. Because several laboratory ratios are correlated, collinearity among them was addressed during feature selection (Section 2.6).

Lead level quartile categorical features were created using quantile-based binning (0%–25%, 25%–50%, 50%–75%, 75%–100%) to identify high-risk thresholds and capture different severity levels of lead exposure. Binary threshold indicators identified patients exceeding WHO standards for elevated blood lead levels (≥0.24 μmol/L or ≥50 μg/L) and Chinese national standards for lead poisoning (≥0.48 μmol/L or ≥100 μg/L), which also served as our primary outcome definition for blood lead re-elevation. The final feature engineering process created additional derived features including medical indicator ratios and polynomial interaction terms, transforming the original clinical variables into an expanded feature set that captures both linear and non-linear relationships in lead toxicity pathophysiology.

### 2.6 Feature selection

Feature selection employed a multi-step approach combining statistical, machine learning, and clinical criteria. Variance inflation factor (VIF) analysis identified and removed 59 highly correlated features (VIF>10) to prevent model instability (Kalnins and Praitis Hill, 2025). Univariate tests (chi-square for categorical, t-tests for continuous variables) identified 34 statistically significant features (p < 0.05) associated with blood lead re-elevation.

Machine learning-based selection methods were then applied, with random forest feature importance (Hu and Szymczak, 2023) ranking features by predictive contribution, Lasso regression with L1 regularization (Daneshvar and Mousa, 2023) selecting 30 features through automatic variable selection, and mutual information analysis (Lei et al., 2023) identifying 35 features capturing non-linear associations with the outcome. We developed an integrated scoring system combining p-values, random forest importance, Lasso coefficients, and mutual information scores, with features weighted by their performance across methods to form the final feature set. The final 36 features were reviewed for clinical plausibility and biological mechanisms, ensuring all selected features had clear clinical interpretations and established roles in lead toxicity pathophysiology. This reduced the initial 142 variables to a final set of 36 clinically meaningful predictors.

### 2.7 Machine learning model development

We implemented and compared three machine learning algorithms based on available computational resources: logistic regression with L2 regularization as the baseline linear model (Li et al., 2022), random forest with optimized hyperparameters (Hu and Szymczak, 2023), and support vector machine with RBF kernel (Huang et al., 2023). The dataset (n = 163) was randomly divided into training (70%, n = 114) and testing (30%, n = 49) sets using stratified sampling (Yates et al., 2023) to maintain the outcome distribution (36.2% re-elevation rate).

Model training employed 5-fold stratified cross-validation (Allgaier and Pryss, 2024) on the training set to assess stability and prevent overfitting. Hyperparameter optimization used randomized search (Rimal et al., 2024) for computational efficiency, with area under the ROC curve (AUC) as the primary optimization metric. To address the moderate class imbalance (1.7:1 ratio), we applied class weighting schemes (Elreedy et al., 2024) to penalize misclassification of the minority class appropriately. All models used a fixed random seed (seed = 42) for reproducibility.

### 2.8 Clinical decision threshold optimization

Recognizing that standard statistical thresholds (0.5) may not be optimal for clinical decision-making, we implemented a cost-sensitive threshold optimization approach (Petrides and Verbeke, 2022). We assigned differential costs to false negatives (missed re-elevation cases) versus false positives (unnecessary intensive monitoring) with a 5:1 ratio, reflecting the clinical priority of preventing missed diagnoses. The optimal threshold was determined by minimizing the total cost function while maximizing clinical utility.

### 2.9 Model evaluation and interpretability

Model performance was evaluated using AUC (Naidu et al., 2023) for overall discriminative ability, sensitivity for ability to identify patients at risk of re-elevation, specificity for ability to correctly identify low-risk patients, precision as positive predictive value, and F1-score as harmonic mean of precision and recall. Clinical metrics included net benefit analysis using decision curve analysis (Vickers and Holland, 2021), number needed to screen for efficiency of risk stratification, and calibration assessment for agreement between predicted and observed probabilities (Badawy et al., 2023).

We interpreted an area under the curve of 0.70–0.80 as acceptable and one above 0.80 as good discrimination, regarded calibration as adequate when the Hosmer-Lemeshow test was non-significant (p > 0.05), and used decision-curve analysis to confirm net clinical benefit across the relevant range of threshold probabilities (Naidu et al., 2023; Nahm, 2022).

We examined feature contributions using Random Forest importance scores (mean decrease in node impurity), feature-value distribution plots, and bar and scatter plots of feature importance. We then analyzed the top 10 features, their dependencies, and individual patient risk profiles to show how features contribute to re-elevation risk in individual patients (Stiglic et al., 2020; Chekroud et al., 2024; Apley and Zhu, 2020).

### 2.10 Statistical analysis

Descriptive statistics were presented as medians (interquartile ranges) for continuous variables and frequencies (percentages) for categorical variables. Between-group comparisons used Mann-Whitney U tests for continuous variables and chi-square tests for categorical variables. Model performance evaluation included ROC curve analysis, confusion matrix assessment, and calibration curve analysis to evaluate prediction accuracy. Feature selection employed variance inflation factor (VIF) analysis, univariate statistical tests, Random Forest feature importance ranking, Lasso regression with L1 regularization, and mutual information analysis with integrated scoring for final feature selection (Nahm, 2022; Habehh and Gohel, 2021). All statistical analyses were performed using Python 3.8 with scikit-learn, pandas, scipy, and matplotlib libraries. Statistical significance was set at p < 0.05 (two-tailed). This study was conducted in accordance with the principles of the Declaration of Helsinki, with patient privacy protected through data de-identification.

## 3 Results

### 3.1 Patient characteristics

A total of 163 patients with confirmed lead poisoning were included in the final analysis after applying inclusion and exclusion criteria. Among these patients, 104 (63.8%) did not experience blood lead re-elevation, while 59 (36.2%) had blood lead re-elevation, defined as blood lead levels returning to ≥0.48 μmol/L during follow-up period after achieving normal levels (<0.48 μmol/L) following chelation therapy.

The cohort also appears broadly representative of lead poisoning in southwestern China. The Chongqing Poison Control Center is a tertiary (Class A) center responsible for poisoning treatment in Chongqing and draws most of its cases from Chongqing, Guizhou and Sichuan. The exposure pattern matched other Chinese reports: adult poisoning was mainly occupational (battery, smelting and paint work) or lifestyle-related, including the inappropriate use of traditional remedies, whereas childhood poisoning was mainly environmental, through hand-to-mouth exposure. The age and sex distribution, with older predominantly male adults and preschool children, agrees with national series and with the male predominance reported for occupational lead exposure elsewhere, including NHANES and European occupational cohorts. Case numbers peaked in 2017 and then fell, mirroring improvements in occupational and environmental control, although childhood cases became relatively more common.

### 3.2 Baseline demographics and clinical characteristics

Regarding clinical symptoms, patients in the re-elevation group showed significantly fewer gastrointestinal symptoms, including less abdominal pain (16.9% vs. 54.8%, p < 0.001), abdominal distension (10.2% vs. 32.7%, p = 0.003), nausea/vomiting (1.7% vs. 15.4%, p = 0.013), decreased appetite (6.8% vs. 24.0%, p = 0.011), constipation (5.1% vs. 20.2%, p = 0.017), and fatigue (8.5% vs. 33.7%, p < 0.001). Hematological parameters showed that the re-elevation group had higher hemoglobin levels (129.0 vs. 115.5 g/L, p = 0.006) and higher lymphocyte percentages (30.6% vs. 27.3%, p = 0.014), but lower neutrophil percentages (60.0% vs. 64.7%, p = 0.005).

### 3.3 Laboratory parameters and treatment variables

The re-elevation group had higher serum calcium (2.4 vs. 2.3 mmol/L, p < 0.001), lower ALT (18.9 vs. 23.4 U/L, p = 0.003) and creatinine (60.1 vs. 68.5 μmol/L, p = 0.016), lower chelation therapy doses (4.0 vs. 9.0 g, p < 0.001), less use of reduced glutathione (49.2% vs. 76.0%, p < 0.001), and lower anemia rates (8.5% vs. 25.0%, p = 0.018).

### 3.4 Model development and performance comparison

Three machine learning algorithms were evaluated for blood lead re-elevation prediction using routinely collected clinical, treatment, and early follow-up data. The dataset consisting of 163 patients was randomly divided into training (70%, n = 114) and testing (30%, n = 49) sets with stratified sampling to maintain class distribution balance across re-elevation cases (36.2%) and non-re-elevation cases (63.8%). Through feature engineering and selection, 36 optimized features were identified from the original 142 clinical variables. All models were trained on standardized features with hyperparameter optimization.

Model performance was assessed using multiple evaluation metrics including accuracy, precision, recall, F1-score, and area under the receiver operating characteristic curve (AUC-ROC). Table 3 presents the performance metrics for all three machine learning models evaluated in this study. Random Forest achieved the highest AUC (0.849) and accuracy (83.7%). Support Vector Machine reached an accuracy of 77.6% and an AUC of 0.801, while Logistic Regression performed less well on all metrics (69.4% accuracy, 0.715 AUC). Random Forest achieved the best precision (77.8%), recall (77.8%), and F1-score (77.8%), indicating high sensitivity in identifying patients at risk of re-elevation, with strong AUC performance.

**TABLE 3:** Model performance comparison on test set.

Model	Accuracy	Precision	Recall	F1-score	AUC
Random forest	0.837	0.778	0.778	0.778	0.849
Support vector machine	0.776	0.667	0.778	0.718	0.801
Logistic regression	0.694	0.565	0.722	0.634	0.715

**FIGURE 3:**
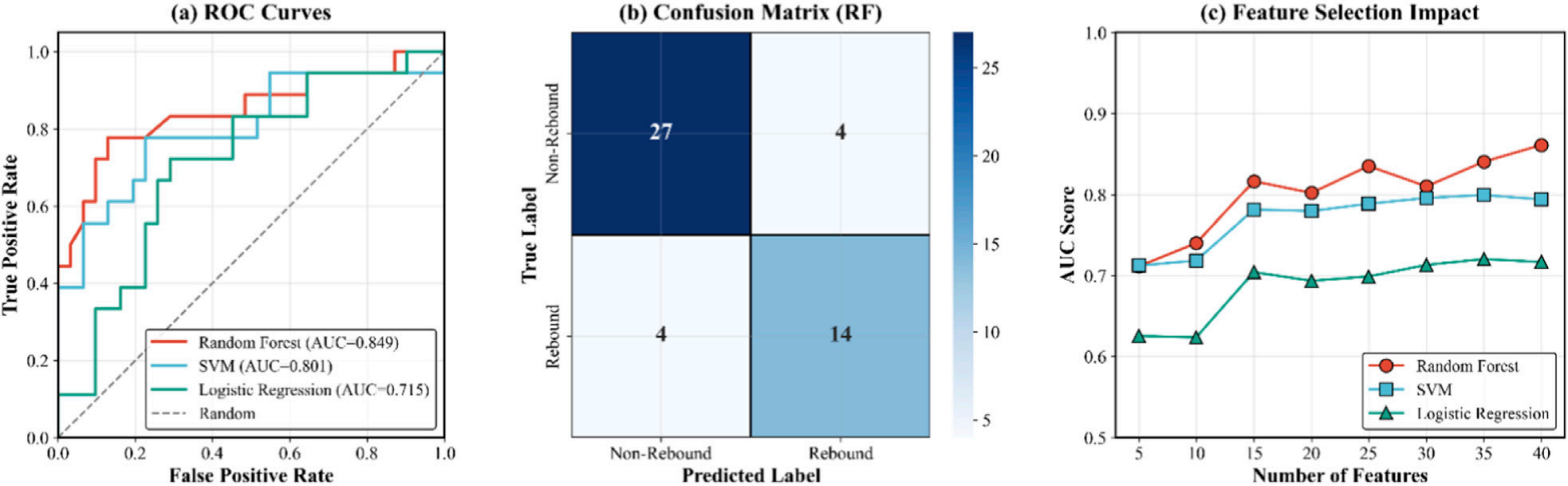
Model performance evaluation and comparison. **(a)** ROC curves comparison showing Random Forest with the highest AUC (0.849). **(b)** Confusion matrix for the Random Forest model (true negatives = 27, false positives = 4, false negatives = 4, true positives = 14). **(c)** AUC across different numbers of features, showing consistent Random Forest performance.

The Random Forest algorithm showed the highest discriminative ability among all tested algorithms, as illustrated in Figure 3a. ROC curve analysis showed Random Forest achieved the highest AUC of 0.849, followed by Support Vector Machine (AUC = 0.801) and Logistic Regression (AUC = 0.715), indicating strong predictive performance for blood lead re-elevation prediction.

### 3.5 Identification of the final model

Based on performance evaluation and clinical applicability, Random Forest was selected as the final prediction model. The decision was informed by multiple factors: first, Random Forest achieved the highest discriminative ability with AUC of 0.849, with clear separation between re-elevation and non-re-elevation cases; second, it had good precision (77.8%), with balanced sensitivity and specificity suitable for clinical use; third, Random Forest’s ensemble design makes it more stable and less prone to overfitting than single classifiers.

The confusion matrix (Figure 3b) showed that Random Forest correctly identified 27 true negatives and 14 true positives, with only 4 false negatives and 4 false positives. The model performance evaluation across varying feature numbers (Figure 3c) showed that Random Forest reached AUC values above 0.80 once at least 15 features were used, with the best performance at 35–40 features.

### 3.6 Clinical utility assessment

#### 3.6.1 Clinical prediction nomogram

**FIGURE 4:**
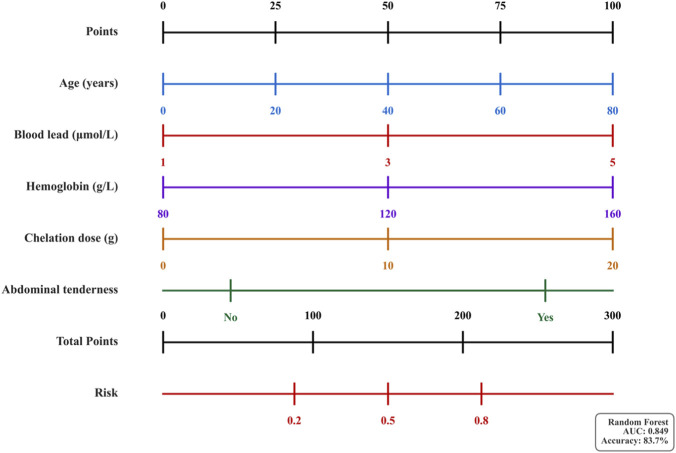
Simplified nomogram-style visualization for blood lead re-elevation risk communication. The figure summarizes five clinically accessible predictors: age (years), blood lead level at admission (μmol/L), hemoglobin (g/L), total chelation therapy dose (g), and abdominal tenderness. It is presented as a visual aid complementary to the full random forest model.

To make the model output easier to interpret, we present a simplified nomogram using five clinically accessible predictors (Figure 4). This figure is intended as a practical communication aid complementary to the full Random Forest model rather than a replacement for that model. The visualization includes age, blood lead level at admission, hemoglobin, total chelation therapy dose, and abdominal tenderness. Each predictor contributes points to a total score, which corresponds to an estimated probability of blood lead re-elevation. This visual tool may help clinicians communicate approximate risk at the bedside while the full model remains the primary analytic approach.

Two de-identified cases illustrate how the nomogram is used. A 10-year-old boy living near a lead-related factory presented with a blood lead of 0.97 μmol/L, hemoglobin 146 g/L, a total chelation dose of 2 g and no abdominal tenderness; his score corresponded to a high predicted probability of re-elevation, and his blood lead subsequently rose from 0.38 to 0.52 μmol/L, confirming recurrence. A 66-year-old woman presented with a blood lead of 2.84 μmol/L, hemoglobin 119 g/L, a total chelation dose of 1 g and no abdominal tenderness; despite the higher admission level her score corresponded to a lower predicted probability, consistent with her stable follow-up. The same five accessible predictors thus translate into individualized risk estimates for a child and for an adult.

#### 3.6.2 Model calibration and clinical utility

**FIGURE 5:**
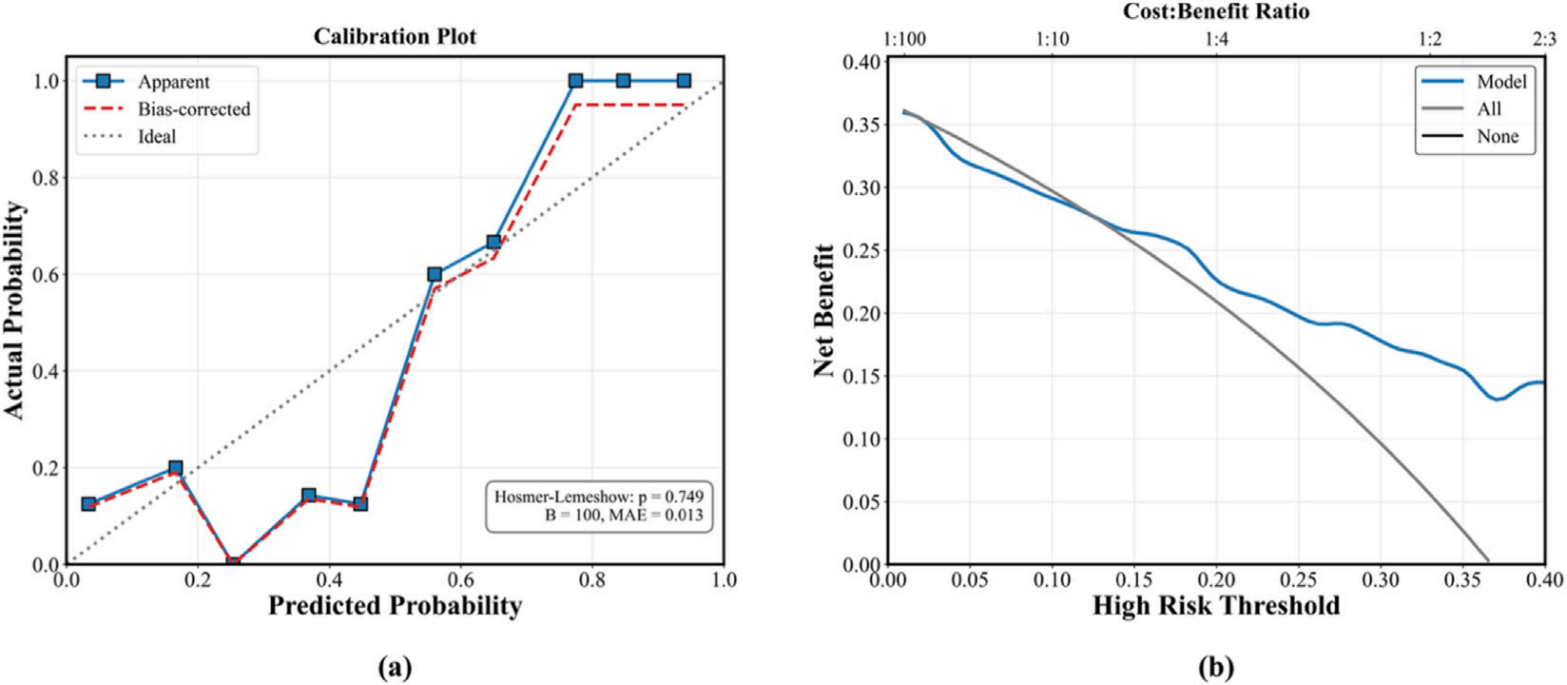
Model calibration and clinical utility assessment. **(a)** Calibration plot showing agreement between predicted and observed probabilities with Hosmer-Lemeshow test results (p-value = 0.749). Bootstrap validation with 100 repetitions showed mean absolute error = 0.013. **(b)** Decision curve analysis showing net benefit across threshold probabilities from 0 to 0.4 with corresponding cost-benefit ratios (1:100, 1:10, 1:4, 1:2, 2:3). The model curve consistently remains above both “Treat All” and “Treat None” strategies, with clinical utility across the range of thresholds tested.

Calibration analysis assessed the agreement between predicted probabilities and observed outcomes (Figure 5a). The calibration plot showed good agreement between predicted and observed probabilities across the range of risk. The Hosmer-Lemeshow test yielded a non-significant p-value of 0.749, indicating adequate calibration. Bootstrap validation with 100 repetitions showed a mean absolute error of 0.013, confirming the model’s reliability in probability estimation. Both apparent and bias-corrected calibration curves closely followed the ideal diagonal line.

Decision curve analysis quantified the clinical utility of the prediction model across different threshold probabilities (Figure 5b). The model provided a clear net benefit compared with “treat all” and “treat none” strategies across the clinically relevant threshold range. The model showed maximum clinical utility at low threshold probabilities, with net benefit exceeding 0.35 when the threshold probability was below 0.05. The model consistently outperformed both the “treat all” and “treat none” strategies across all thresholds tested. The corresponding cost-benefit ratios ranged from 1:100 to 2:3, indicating the model’s value across various clinical decision-making contexts and resource allocation scenarios.

### 3.7 Model explanation

To address the “blackbox” nature of machine learning models, we performed model interpretation using Random Forest’s feature importance scores and analyzed individual patient predictions for representative low-risk (15.0% probability) and high-risk (83.0% probability) cases.

#### 3.7.1 Global model explanation

**FIGURE 6:**
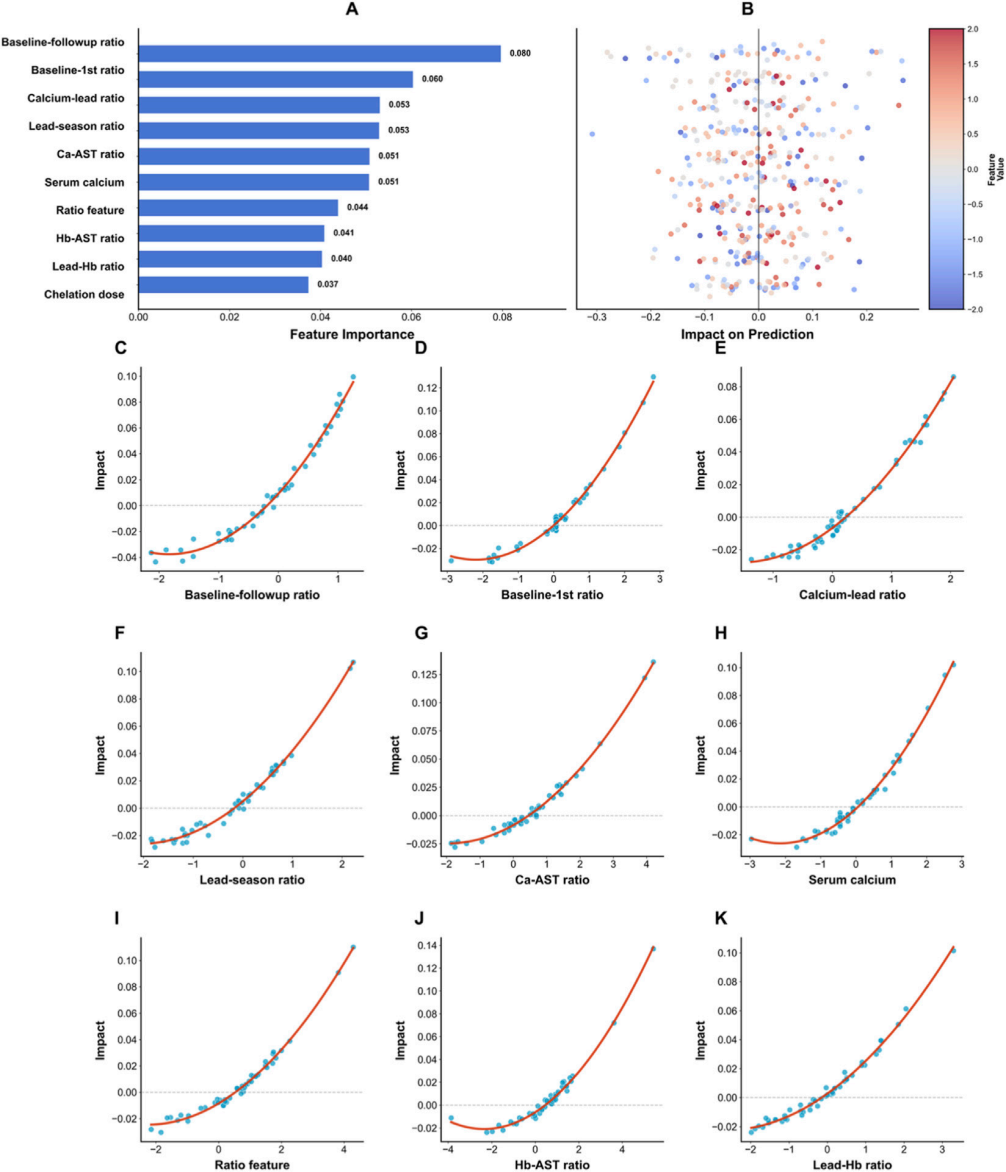
Global model explanation by feature importance analysis. **(A)** Feature importance bar plot showing the top 10 predictive features with importance values. **(B)** Feature value distribution plot showing the impact of each feature on model predictions, with colors indicating feature values (red: higher, blue: lower). **(C–K)** Feature dependence plots showing non-linear relationships between individual features and prediction impact.

Feature importance analysis (Figure 6A) identified key predictive features created through feature engineering. The baseline-to-followup blood lead ratio emerged as the strongest predictor (importance = 0.080), capturing lead clearance dynamics following chelation therapy.

The baseline-to-first followup ratio (importance = 0.060) provides insights into early treatment response. The calcium-lead ratio (importance = 0.053) and lead-season ratio (importance = 0.053) pointed to calcium-lead competitive inhibition and to seasonal variation in exposure.

The calcium-to-AST ratio (importance = 0.051) and serum calcium (importance = 0.051) captured multi-system toxicity patterns. The hemoglobin-to-AST ratio (importance = 0.041) and lead-hemoglobin ratio (importance = 0.040) reflected hematological involvement in lead toxicity.

Chelation dose (importance = 0.037) links treatment intensity to re-elevation risk and supports giving adequate initial chelation.

Figure 6B shows that feature values were unevenly distributed across patients, with clusters that suggest distinct risk phenotypes. The dependence plots (Figures 6C–K) show non-linear relationships between predictors and re-elevation risk, particularly for ratio features where the relationship follows a characteristic curve consistent with dose-response patterns in heavy metal toxicity.

#### 3.7.2 Local model explanation

**FIGURE 7:**
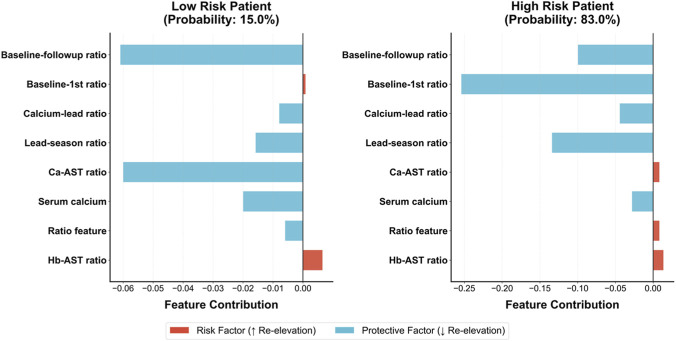
Local model explanation showing individual patient risk profiles. Left panel shows a low-risk patient (predicted probability 15.0%) where most features contribute as protective factors. Right panel shows a high-risk patient (predicted probability 83.0%) where multiple risk factors contribute to higher re-elevation probability. Feature contributions are color-coded: red bars indicate risk factors increasing re-elevation probability, cyan bars indicate protective factors decreasing risk.

Individual patient analysis (Figure 7) shows personalized risk assessment using the top 8 most important features. The low-risk patient (15.0% probability) shows protective contributions from baseline-followup ratio, Ca-AST ratio, serum calcium, and lead-season ratio (cyan bars), indicating good treatment response and favorable calcium homeostasis. Minor risk factors include baseline-1st ratio and Hb-AST ratio (red bars).

The high-risk patient (83.0% probability) presents multiple converging risk factors including elevated Ca-AST ratio and Hb-AST ratio (red bars). Several protective factors remain present, including baseline-1st ratio, lead-season ratio, and serum calcium (cyan bars), suggesting that intensive intervention targeting specific risk factors may still be beneficial.

These contrasting profiles illustrate how the model integrates multiple biological pathways to stratify patients. For high-risk patients, the model identifies specific intervention targets enabling evidence-based, individualized care plans.

### 3.8 Convenient application for clinical utility

The final prediction model was implemented as a prototype web application for research use. When clinicians enter the required clinical features, the application automatically predicts the risk of blood lead re-elevation for individual patients. The application also displays feature contributions showing which factors increase or decrease re-elevation risk for each patient, supporting risk communication and hypothesis generation. The web application is accessible online at https://lead-poisoning-prediction-system-mlxchfxnvf2bfpnkkl2ay2.streamlit.app/. It should not be used as a stand-alone clinical decision tool without further external validation.

## 4 Discussion

To our knowledge, this is the first study to investigate machine learning approaches for blood lead re-elevation prediction following chelation therapy. While previous studies have developed prediction models for initial blood lead elevation in community screening settings (Potash et al., 2020; Mulhern et al., 2022; Abhadiomhen et al., 2024), our work addresses the distinct clinical challenge of identifying patients at risk for post-treatment recurrence. The Random Forest model showed the highest discriminative ability (AUC = 0.849), outperforming Support Vector Machine (AUC = 0.801) and Logistic Regression (AUC = 0.715). Our model showed higher internal discrimination than the Mulhern et al. model for initial blood lead elevation (AUC = 0.792); because that model addressed a different outcome and population and was not evaluated on our data, this is an indirect comparison rather than a head-to-head benchmark. Our final model incorporated 36 optimized features selected from 142 original clinical variables and is best interpreted as a tool for post-treatment risk assessment using routinely collected in-hospital and early follow-up information rather than as a purely admission-time model.

The substantially higher re-elevation rate in children (52.3%) compared with adults (30.3%) reflects children’s greater vulnerability, through higher absorption and ongoing neurodevelopment (Liu et al., 2014; Albores-Garcia et al., 2021; Assi et al., 2016). Relying on baseline blood lead alone has limited ability to identify who will re-elevate. The model integrates additional routinely collected information and reached 77.8% sensitivity in internal testing; this is not a formal comparison against a single-variable baseline and should be confirmed prospectively.

The baseline-to-followup ratio emerged as the strongest predictor (importance = 0.080), capturing treatment response dynamics. The baseline-to-first-followup and calcium-lead ratios also ranked highly. Their prominence is consistent with a contribution from early treatment response and from nutritional and multi-organ factors to susceptibility to lead toxicity (Assi et al., 2016).

Several of the most influential features have clear pathophysiological interpretations, whereas others should be regarded as model-derived associations that require confirmation. The baseline-to-follow-up blood lead ratio, the strongest predictor, captures the dynamics of lead clearance after chelation, and the total chelation dose reflects treatment adequacy, both consistent with the clinical observation that under-treatment predisposes to re-elevation. The hemoglobin-to-AST and calcium-to-AST ratios most likely reflect the degree of multisystem (hematologic, hepatic and mineral-metabolism) involvement. By contrast, the prominence of the calcium-to-lead and blood lead-to-season ratios was not corroborated by conventional statistical analysis in our prior study of this cohort, in which neither low calcium nor season was significantly associated with re-elevation; these features should therefore be interpreted as hypotheses generated by the model rather than as established clinical predictors, to be tested in future work.

The misclassified cases are also informative. All four false negatives (patients who re-elevated but were predicted to be low risk) were adults; they tended to have relatively high admission blood lead but otherwise did not stand out on the features the model weights most heavily, so re-elevation driven mainly by endogenous bone-lead release or incomplete source removal can be missed when early clearance looks adequate. Most false positives, by contrast, were children with comparatively low admission blood lead who were flagged as high risk but did not re-elevate, plausibly because of adequate treatment and closer, caregiver-supported follow-up. Misclassification was proportionally more common in children than in adults, so pediatric predictions in particular should be read alongside clinical judgment. Both patterns fit the toxicokinetic differences between children and adults and confirm that the model is an adjunct to, not a substitute for, clinical assessment.

The model uses routinely collected clinical data and may help move follow-up planning toward a more personalized, risk-based approach. Given that current CDC and AAP guidelines lack specific risk assessment tools and many patients show poor follow-up adherence, our findings suggest that this type of model could help clinicians identify higher-risk patients who may benefit from intensified monitoring. However, implementation should be cautious until the model is externally validated and its performance is confirmed in broader populations.

### 4.1 Limitations

Several limitations should be acknowledged. First, this was a retrospective single-center study with a modest sample size, which increases the risk of selection bias and model overfitting. Second, evaluation was limited to an internal train-test split and bootstrap-based calibration assessment; therefore, the model requires temporal and external validation before routine clinical use. Third, some influential predictors reflected treatment course and early follow-up measurements, so the present model should be interpreted as supporting post-treatment monitoring rather than purely admission-time triage. Finally, the inclusion of both pediatric and adult patients improves clinical coverage but also introduces heterogeneity that should be examined in larger multicenter datasets.

In addition, because post-discharge blood lead monitoring was sparse, we could not reliably quantify the time to re-elevation, and we therefore do not report a formal time-to-event (survival) analysis; the timing of recurrence should be characterized in prospective studies with scheduled monitoring. The use of a single model spanning children and adults, although it maximizes statistical power, does not capture age-specific toxicokinetic differences, and age-stratified models should be developed in larger multicenter cohorts. Finally, the model underwent internal evaluation only; external and temporal validation are required before clinical use.

Two further points concern the outcome and the predictors. The early follow-up blood lead measurements that feed the most influential composite features were obtained during in-hospital treatment monitoring and the early post-treatment period, before any re-admission; they therefore precede and are independent of the re-admission events used to define the outcome and do not encode it. Conversely, because re-elevation was ascertained through re-admission, patients who re-elevated but did not return for care may be under-ascertained, so re-admission is a specific but conservative marker of clinically significant re-elevation; prospective studies with scheduled monitoring are needed to capture silent re-elevation.

## 5 Conclusion

This study presents, to our knowledge, the first machine-learning model developed specifically to predict blood lead re-elevation after chelation therapy, achieving an AUC of 0.849 and an accuracy of 83.7% on internal testing. Unlike existing models that predict initial blood lead elevation in community populations, it addresses the clinically distinct problem of post-treatment recurrence in hospitalized patients. Compared with current practice, in which all patients are advised to undergo the same fixed-interval blood lead monitoring after discharge (an approach undermined by poor adherence, especially among the many asymptomatic patients), the model provides an individualized re-elevation probability that could help concentrate monitoring on higher-risk patients while reducing unnecessary testing in lower-risk patients, given the higher re-elevation rate observed in children than in adults (52.3% vs. 30.3%). These findings provide a basis for prospective and external validation studies aimed at improving follow-up care and the early detection of high-risk patients.

## Data Availability

The data analyzed in this study is subject to the following licenses/restrictions: The dataset contains retrospective clinical data from lead poisoning patients and includes sensitive health information. Due to patient privacy, institutional ethics requirements, and data protection regulations, the raw dataset is not publicly available. De-identified data may be made available by the corresponding authors upon reasonable request and with approval from the relevant institution and ethics committee. Requests to access these datasets should be directed to xiaoguier0610@126.com.
